# Tale of two assays: Comparison of modern donor-derived cell-free DNA technologies

**DOI:** 10.1016/j.jhlto.2024.100090

**Published:** 2024-04-01

**Authors:** Brian Hsi, Johanna Van Zyl, Komal Alam, Hira Shakoor, Dana Farsakh, Amit Alam, Shelley Hall

**Affiliations:** a Baylor University Medical Center, Dallas, Texas; b New York University School of Medicine, New York, New York

**Keywords:** heart transplantation, acute allograft rejection, acute cellular rejection, antibody-mediated rejection, donor-derived cell-free DNA

## Abstract

Little is known about the comparative differences between the Allosure (CareDx) and Prospera (Natera) donor-derived cell-free DNA (dd-cfDNA) assays following heart transplantation. We retrospectively analyzed 248 consecutive samples that had both dd-cfDNA assays simultaneously performed. Twenty-six biopsy specimens were available within 7 days from dd-cfDNA assays. Both dd-cfDNA assays were correctly suggestive of rejection when biopsy was available. However, discordant classifications were present in 23/248 samples when utilizing respective recommended cutoff values for each assay (0.12% for Allosure and 0.15% for Prospera). Discordance was due to increased classification as abnormal results with Allosure (McNemar’s *p* = 0.004). However, there were no significant differences between assays when identical thresholds of 0.12% or 0.15% were implemented for both assays (McNemar’s, *p* = non-significant). We conclude that both dd-cfDNA assays can be utilized interchangeably for surveillance of rejection following heart transplantation.

## Background

The diagnosis of rejection remains challenging with the gold standard of an endomyocardial biopsy (EMBx) remaining an integral part of protocol surveillance monitoring following heart transplantation (HT).^1^ Despite standardized histopathological criteria for rejection, the procedure has limitations due to its invasive nature, potential complications, costs, as well as significant inter-observer variability in the histological assessment.^2^, ^3^

Contemporary noninvasive techniques, namely donor-derived cell-free DNA (dd-cfDNA), utilize single-nucleotide polymorphisms (SNP) across the genome to distinguish between recipient and donor molecules with an increase in dd-cfDNA levels at times of acute cellular rejection (ACR) and antibody-mediated rejection (AMR) or any other significant injury to the donor graft.^3^ Prior studies have validated 2 different dd-cfDNA assays, namely Allosure (CareDX; Brisbane, CA) and Prospera (Natera; Austin, TX).^4^, ^5^ As a result, HT programs are reducing the number of routine surveillance EMBx and placing greater emphasis on noninvasive rejection monitoring.^1^, ^6^, ^7^, ^8^

In a recent study comparing the 2 dd-cfDNA technologies, there was no significant difference in the detection of acute rejection.^9^ However, the study reported its findings with the cutoff valves of 0.15% for both assays despite different thresholds utilized by their respective validation studies. Interpretive guides published for Allosure and Prospera utilize respective thresholds of 0.12% and 0.15%, and circumstances in which dd-cfDNA levels below those thresholds are unlikely to represent graft injury and rejection. Herein, we report a real-world analysis of agreement between both dd-cfDNA technologies utilizing manufacturer-suggested thresholds (0.12% vs 0.15%) as well as identical thresholds (0.12% vs 0.12% and 0.15% vs 0.15%).

## Methods

This is a retrospective observational study comparing results from paired Allosure and Prospera dd-cfDNA assays from OHT recipients between January 2022 and April 2023. Consecutive paired, same-day dd-cfDNA results and EMBx results, when available within 7 days from collection of dd-cfDNA samples, were included. Concordance between assays was assessed utilizing thresholds of ≥0.12% for Allosure and ≥0.15% for Prospera indicative of an abnormality,^4^, ^5^ as well as using identical thresholds (≥0.12% vs ≥0.12% and ≥0.15% vs ≥0.15%) for both assays. The study was approved by our Institutional Review Board.

### Data collection and definitions

Data were collected using chart review. Severe early graft dysfunction was defined by mechanical support within 24 hours of HT. Rejection was defined if T-cell mediated rejection (ACR ≥ 2R) and/or AMR (pathological antibody-mediated rejection (pAMR) ≥ 1) were detected on EMBx. Rejection and mortality were collected following the first dd-cfDNA sample. Cardiac allograft vasculopathy (CAV > 0) was assessed using coronary angiography.

### Rejection surveillance protocol

Dd-cfDNA and donor specific antibodies are tested monthly during year 1, quarterly during years 2 to 3, biannually during years 4 to 5, then annually thereafter or per clinical concern. Due to concerns for elevated dd-cfDNA levels from preservation and ischemia-reperfusion injury early after transplantation, we do not routinely obtain dd-cfDNA earlier than 1 month after transplant at our institution. Additionally, rejection surveillance includes obtaining EMBx and the Molecular Microscope Diagnostic System, echocardiogram, and right heart catheterization at 2 weeks, 6 weeks, and 1 year post-transplant or per clinical concern. CAV is assessed at week 6 and 1-year post-transplant.

### Summary of immunosuppression protocol

At our institution, the decision to undergo induction therapy with antithymocyte globulin is based on patient’s immunologic risk category, which is defined by the donor-specific antibody profile during crossmatch, computed panel reactive antibodies, and if the patient underwent desensitization therapies before transplantation. Otherwise, the standard perioperative immunosuppressive regimen is methylprednisolone and mycophenolate mofetil. Maintenance immunosuppressive regimen generally consists of calcineurin inhibitors (preferred agent is tacrolimus with target drug levels according to time from transplant; 0-3 months: 12-15 ng/ml, 3-6 months: 10-12 ng/ml, 6-12 months: 8-10 ng/ml, >12 months: 5-8 ng/ml), cell cycle inhibitors (with mycophenolate mofetil as the preferred agent, dosed at 1000 mg twice daily unless weight <60 kg, age > 60 years, and white blood cell count < 4,000, circumstances under which the dose would be decreased to 500 mg twice daily), and corticosteroids, which is usually tapered and discontinued by 6 months post-transplant.

### Statistical analysis

Agreement of dd-cfDNA results between assays was quantified as Cohen’s kappa and percentage of agreement. Cohen’s kappa (κ) was interpreted as: <0, no agreement; 0 to 0.20, slight, 0.21 to 0.40 fair, 0.41 to 0.60 moderate, 0.61 to 0.80 substantial, and 0.81 to 1.00 almost perfect.^5^ McNemar’s test was used to evaluate systematic differences. All analyses were performed in R (version 4.2).

## Results

**Figure 1:**
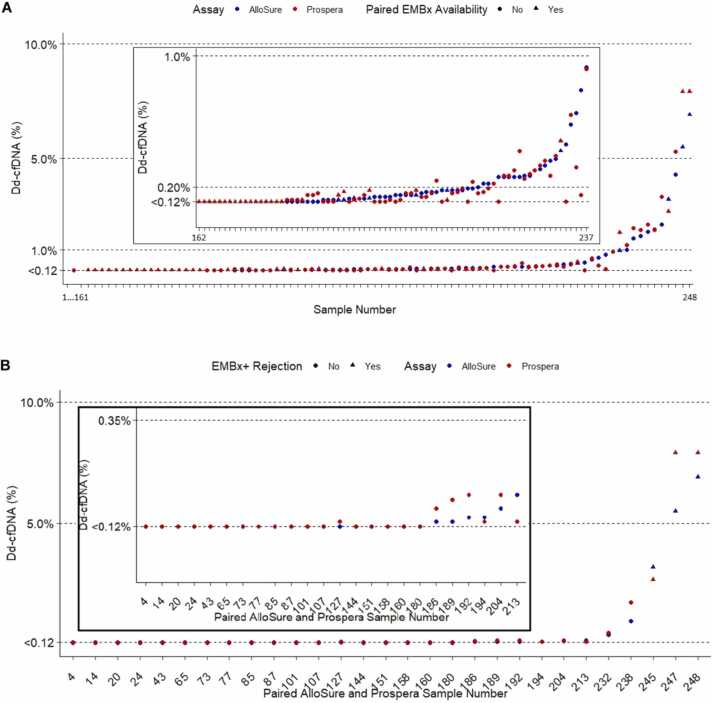
**(A)** Absolute values of paired dd-cfDNA samples. **(B)** Paired dd-cfDNA samples associated with endomyocardial biopsies.

Two hundred and forty-eight corresponding samples obtained on the same day and analyzed to quantify % dd-cfDNA using the Allosure and Prospera assays were included from 46 heart transplant recipients. Ten dd-cfDNA results were excluded where samples were obtained on consecutive days. The median days after transplantation at the time of study enrollment were 71 (inter-quartile range (IQR) 43-95). Timing of dd-cfDNA samples ranged between 18 and 506 days with a median time post-HT of 163 days [IQR: 99-255]. Patient characteristics, reported in Table 1, included a median age of 59 years, 70% were male, and 24% had ischemic cardiomyopathy. Figure 1A shows the values of each paired dd-cfDNA. Figure 1B specifically delineates the dd-cfDNA samples that are associated with a paired EMBx. Agreement between both dd-cfDNA assays utilizing their cutoff values of 0.12% for Allosure and 0.15% for Prospera was substantial at 91% (κ = 0.73, *p* < 0.001) (Table 2). Among 9% of discordant samples classifications, there were significant systematic differences where Allosure was more likely to classify a sample as abnormal compared to Prospera (McNemar’s *p* = 0.004). Paired EMBx results were available in 26 samples including 1 case of ACR = 3R and 2 cases of pAMR1 rejection. In the cases of rejection, both assays had elevated dd-cfDNA. Agreement among the samples with a paired negative EMBx was 92% with 18 of 26 (69%) Allosure dd-cfDNA <0.12% and 20 of 26 (77%) Prospera dd-cfDNA <0.15%.

**Table 1:** Patient Characteristics and Outcomes

*Patient characteristics*	*Overall (n = 46)*
Age (years), median [IQR]	59 [52, 63]
Body mass index (BMI), kg/m^2^, median [IQR]	28 [25, 32]
Sex, male, *n* (%)	32 (70%)
Race, *n* (%)	
Asian	1 (2%)
Black or African American	17 (37%)
White	26 (57%)
African American and White	1 (2%)
Not reported	1 (2%)
Hispanic ethnicity, *n* (%)	5 (11%)
HF etiology, *n* (%)	
ICM	11 (24%)
NICM	33 (72%)
Mixed ICM/NICM	2 (4%)
UNOS status, *n* (%)	
1	0 (0%)
2	17 (37%)
3	10 (22%)
4	15 (33%)
5	0 (0%)
6	4 (9%)
Calculated panel reactive antibodies (%) at HT, median [IQR]	22 [0, 63]
Induction	
None	37 (80%)
Simulect/Basiliximab	2 (4%)
ATG	7 (15%)
*Outcomes post-HT*	
Severe early graft dysfunction requiring mechanical support, *n* (%)	5 (11%)
CAV > 0 at baseline, *n* (%)	N = 41
	1 (2%)
CAV > 0 at 1 year, *n* (%)	N = 31
	3 (10%)
Rejection	4 (9%)
Death	2 (4%)

Abbreviations: ATG, anti-thymocyte globulin; CAV, cardiac allograft vasculopathy; HT, heart transplantation; ICM, ischemic cardiomyopathy; IQR, inter-quartile range; NICM, non-ischemic cardiomyopathy; UNOS, United Network for Organ Sharing.

**Table 2:** Agreement Between Allosure and Prospera Assays Comparing dd-cfDNA Above and Below 0.12% for Allosure with cfDNA Above and Below 0.15% for Prospera

Cohort	Rejection surveillance results				% Agreement	Cohen’s kappa coefficient (κ)	McNemar’s *p*-value
Overall	Prospera	Allosure			91%	0.73	0.004
		<0.12%	≥0.12%	Total			
	<0.15%	181	19	190			
	≥0.15%	4	44	48			
	Total	185	63	248			
Paired biopsy-negative	Prospera	Allosure			92% (24/26)		
		<0.12%	≥0.12%	Total			
	<0.15%	18	2	20			
	≥0.15%	0	6	6			
	Total	18	8	26			
Paired biopsy with rejection	Prospera	Allosure			100% (3/3)		
		<0.12%	≥0.12%	Total			
	<0.15%	0	0	0			
	≥0.15%	0	3	3			
	Total	0	3	3			

Abbreviations: ACR, acute cellular rejection; dd-cfDNA, donor-derived cell-free DNA.

Rejection on biopsy was defined as ACR ≥ 2R or pAMR > 0.

In Table 3, agreement between the assays was evaluated using the same threshold. At the 0.12% and 0.15% thresholds, agreement remains substantial at 92% (κ = 0.79, *p* < 0.001) and 93% (κ = 0.78, *p* < 0.001), respectively. However, when using these same thresholds, there are no significant systematic differences between assays (McNemar’s *p* = 0.50 and *p* = 1.00, respectively) with approximately half of discordant results suggestive of an abnormality for either assay at ≥0.12% (12/20 discordant results with Allosure ≥0.12% vs 8/20 with Prospera ≥0.12%) or at ≥0.15% (9/17 discordant results with Allosure ≥0.15% vs 8/17 with Prospera ≥0.15%).

**Table 3:** Agreement Between Allosure and Prospera Assays Comparing dd-cfDNA Above and Below 0.12% and 0.15%

Cohort	Rejection surveillance results				% Agreement	Cohen’s kappa coefficient (κ)	McNemar’s *p*-value
Overall	Prospera	Allosure			92%	0.78	0.50
		<0.12%	≥0.12%	Total			
	<0.12%	177	12	189			
	≥0.12%	8	51	59			
	Total	185	63	248			
Paired biopsy-negative	Prospera	Allosure			96% (25/26)		
		<0.12%	≥0.12%	Total			
	<0.12%	17	0	17			
	≥0.12%	1	8	9			
	Total	18	8	26			
Paired biopsy with rejection	Prospera	Allosure			100% (3/3)		
		<0.12%	≥0.12%	Total			
	<0.12%	0	0	0			
	≥0.12%	0	3	3			
	Total	0	3	3			
Overall	Prospera	Allosure			93%	0.78	1.00
		<0.15%	≥0.15%	Total			
	<0.15%	191	9	200			
	≥0.15%	8	40	48			
	Total	199	49	248			
Paired biopsy-negative	Prospera	Allosure			85% (22/26)		
		<0.15%	≥0.15%	Total			
	<0.15%	19	1	20			
	≥0.15%	3	3	6			
	Total	22	4	26			
Paired biopsy with rejection	Prospera	Allosure			100% (3/3)		
		<0.15%	≥0.15%	Total			
	<0.15%	0	0	0			
	≥0.15%	0	3	3			
	Total	0	3	3			

Abbreviations: ACR, acute cellular rejection; dd-cfDNA, donor-derived cell-free DNA.

Rejection on biopsy was defined as ACR ≥ 2R or pAMR > 0.

Overall, 4 patients experienced rejection and 2 deaths were recorded (Table 4). The fourth rejection case was persistent AMR despite treatment for rejection with 3 consecutive positive pAMR1 results over the course of 3.5 months with elevated dd-cfDNA on both assays ranging between 2% and 18.4%.

**Table 4:** Biopsy-Proven Rejection Events up to 1 Year Following the First Included dd-cfDNA Result With the Closest dd-cfDNA Results

Subject	Rejection	Allosure dd-cfDNA, time relative to EMBx	Prospera dd-cfDNA, time relative to EMBx	Vital status
1	ACR = 3R	3.2%, 0 day	2.7%, 0 day	Deceased
2	pAMR1	5.5%, 0 day	7.9%, 0 day	Alive
3	ACR = 2R	0.26%, 1 day prior	0.42%, 0 day	Alive
4	pAMR1	5.4%, 23 days prior	2.1%, 23 days prior	Deceased

Abbreviations: ACR, acute cellular rejection; dd-cfDNA, donor-derived cell-free DNA; EMBx, endomyocardial biopsy; pAMR, pathological antibody-mediated rejection.

## Discussion

Our study found significant agreement between the Allosure and Prospera dd-cfDNA assays, and both were correctly suggestive of presence or absence of rejection when compared to EMBx as the reference standard. These results were concordant with those published by Rodgers et al.^9^ Although there were differences in assay discrimination when utilizing manufacturer-suggested thresholds (0.12% for Allosure and 0.15% for Prospera), this discrepancy was no longer present when using identical thresholds for differentiating presence versus absence of rejection. Although there may be other applications that may take advantage of the differences between the two assays (namely, the utilization of a 405 SNP panel by Allosure versus a 13,292 SNP panel by Prospera), our study suggests that, in clinical practice with the sole intention of identifying the presence of allograft rejection, both assays appear interchangeable without clinically meaningful differences between them.

There are multiple limitations to this study. First, it is a single-center, retrospective study and may not reflect experiences of other centers. Second, the modest size of our cohort was only sufficient to determine the concordance between the standard and expanded SNP assays. While both assays were correctly suggestive of the presence or absence of rejection in our study when compared to EMBx as the reference standard, the size of our cohort as well as the number of corresponding EMBx performed do not allow for adequate assessment of each assay’s ability to correctly identify rejection, although other studies have examined this issue in greater detail.^4^, ^5^ Larger studies are needed to validate these preliminary findings.

## Disclosure statement

The authors declare the following financial interests/personal relationships which may be considered as potential competing interests: Dr Alam has been a speaker for both Caredx, Abbott, and was previously the Private Investigator for Natera’s ProTECT trial. Dr Hall has served as a consultant/advisor for Abott, Abiomed, Evaheart, Natera, and Caredx. All other authors have reported that they have no relationships relevant to the contents of this paper to disclose. All other authors declare that they have no known competing financial interests or personal relationships that could have appeared to influence the work reported in this paper.
